# Longitudinal Predictors of Self-Rated Health and Mortality in Older Adults

**DOI:** 10.5888/pcd11.130241

**Published:** 2014-06-05

**Authors:** Diane C. Wagner, Jerome L. Short

**Affiliations:** Author Affiliation: Jerome L. Short, George Mason University, Fairfax, Virginia.

## Abstract

**Introduction:**

Few studies have compared the effects of demographic, cognitive, and behavioral factors of health and mortality longitudinally. We examined predictors of self-rated health and mortality at 3 points, each 2 years apart, over 4 years.

**Methods:**

We used data from the 2006 wave of the Health and Retirement Study and health and mortality indicators from 2006, 2008, and 2010. We analyzed data from 17,930 adults (aged 50–104 y) to examine predictors of self-rated health and data from a subgroup of 1,171 adults who died from 2006 through 2010 to examine predictors of mortality.

**Results:**

Time 1 depression was the strongest predictor of self-rated health at all points, independent of age and education. Education, mild activities, body mass index, delayed word recall, and smoking were all associated with self-rated health at each point and predicted mortality. Delayed word recall mediated the relationships of mild activity with health and mortality. Bidirectional mediation was found for the effects of mild activity and depression on health.

**Conclusion:**

Medical professionals should consider screening for depression and memory difficulties in addition to conducting medical assessments. These assessments could lead to more effective biopsychosocial interventions to help older adults manage risks for mortality.

## Introduction

Although medical advances, improved nutrition, and reduced mortality in early life have produced life expectancies extending beyond those of previous generations, recent evidence indicates that baby boomers have higher rates of chronic disease than members of the previous generation (1), suggesting that lifestyle factors affect health in late life. In addition, age of death decreased for women in 42.8% of US counties from 1992 to 2006 (2), and in a nationally representative UK sample, not smoking, engaging in regular physical activity, consuming alcohol moderately, and obtaining adequate vitamin C (an indicator of fruit and vegetable intake) were associated with lower risk of mortality and lowered the all-cause mortality rate by 58% (3). Memory functioning, and especially mild cognitive impairment (MCI) is often not included in survival analyses, but it is an important predictor of mortality. A review of dementia, cognitive impairment, and mortality in international and US community populations found a positive association between increasing levels of severity of cognitive impairment and mortality. Mortality risk increased with mild levels of impairment (4). MCI is associated with increased risk for dementia and mortality, and increased mortality risk in MCI is likely due to dementia and dementia-related illnesses (5). Memory impairment in Alzheimer’s disease (AD) may increase risk for mortality due to the complications such as immobility, swallowing disorders, malnutrition, and pneumonia (6). Rather than being a precursor to AD, age-related memory loss is a distinct process that probably results from changes in neural functioning rather than loss of neurons, as is the case with AD (7). Few studies of mortality in community samples have examined the effect of MCI on mortality (8). 

Delayed word recall (DWR) is among the most sensitive measures of memory functioning. In a study of 3,778 older adults without dementia, 3-word DWR predicted mortality at 10 years, with a 52% greater mortality in those who remembered 0 words compared with those who remembered all 3 words (4). High depressive symptoms predicted mortality in older adults after controlling for sociodemographic factors, disease, biological, and behavioral risk factors and suggested that motivational depletion (eg, everything I did was an effort) may help explain the effect of depressive symptoms on mortality (9).

The purpose of this study was to examine demographic, cognitive, behavioral, and biological risk and protective factors of self-rated health and mortality longitudinally in a nationally representative sample. Our research questions were 1) How well do age, education, recall memory, depression, physical activity, sleep, and body mass index (BMI) at time 1 predict self-rated health (SRH) 2 and 4 years later, and 2) How well do education, recall memory, depression, physical activity, sleep, BMI, and smoking predict mortality.

## Methods

### Participants

We used data from 17,930 adults in the 2006, 2008, and 2010 waves of the Health and Retirement Study (HRS) and 1,319 adults in the study who died from 2006 through 2010. The HRS is an ongoing biennial survey of a nationally representative US sample of adults aged 50 and older designed to examine health and retirement of adults in the community. The HRS (10) is sponsored by the National Institute on Aging and is conducted by the University of Michigan. Details of HRS recruitment, participant characteristics, and interviewing methods have been described elsewhere (11).

### Measures

Demographic characteristics were age, sex, and years of education from 2006 HRS data. Depression was measured with an 8-item version of the Centers for Epidemiological Study Depression Scale (CES-D) (12,13). All 8 items were summed and ranged from 0 to 8; higher numbers indicated more depressive symptoms. Frequency of mild activities was assessed with 1 item that asked, “How often do you take part in sports or activities that are mildly energetic, such as vacuuming, laundry, or home repairs?” Responses were 1 = hardly ever or never, 2 = 1 to 3 times a month, 3 = once a week, 4 = more than once a week, and 5 = every day. Restful sleep was assessed with 1 item that asked, “How often do you feel really rested when you wake up in the morning?” Responses were 1 = rarely or never, 2 = sometimes, and 3 = most of the time. We computed BMI by using participants’ height (m^2^) and weight (kg). Recall memory was assessed with DWR, which was the number of words recalled in any order from a list of 10 nouns from the Established Populations for Epidemiologic Study of the Elderly in Iowa (14,15). Smoking was assessed with 1 item that asked, “Do you smoke cigarettes now?” Chronic pain was measured with 1 item that asked, “Are you often troubled with pain?” Chronic disease included items indicating whether a doctor had ever informed a participant that he or she had high blood pressure, heart disease, diabetes, stroke, cancer, lung disease, or arthritis. SRH was measured in the 2006, 2008, and 2010 HRS waves with a self-rated perceived health item that correlated with all physical health indicators (15) and asked, “Would you say your health is excellent, very good, good, fair, or poor?” Responses were 1 = poor, 2 = fair, 3 = good, 4 = very good, and 5 = excellent. Mortality was calculated with the month and year of death.

### Data analyses

We performed multiple imputation of missing data using Mplus, version 7.1 (Muthen and Muthen, Los Angeles, California) and data analyses using SPSS, version 19.0 (SPSS, Inc, Chicago, Illinois). We conducted a 1-way analysis of variance to compare the percentage of obese (BMI ≥30.0) and nonobese (BMI <30.0) people with specific chronic conditions. To test predictors of self-rated health and mortality, we performed bivariate correlations. Variables that were significantly associated with SRH and mortality at *P* < .10 were included in the regression models, and significance was set at *P* < .05. To evaluate predictors of SRH at 3 points, we conducted hierarchical multiple linear regressions, with demographic variables of age and years of education as explanatory variables in model 1. In model 2, we added the significantly correlated predictors: DWR, depression, sleep, mild activities, smoking, and BMI. Significance tests for change in *R* ^2^ were conducted to assess the degree to which additional variables accounted for the variance in SRH independent of age and education.

To evaluate predictors of mortality, a Cox regression analysis was conducted with education, SRH, DWR, depression, sleep, mild activities, smoking, and BMI entered as a set. Age was modeled as a time-dependent predictor. Because 2 predictors violated the proportional hazards assumption (SRH and mild activities), we used a segmented, time-dependent Cox regression model.

Because of the strength and consistency of the relationship of DWR with outcomes of health and mortality in the regression models, we conducted additional multiple linear regressions to identify predictors of DWR and entered education, BMI, smoking, and mild activities as a set. We tested several mediating relationships of predictors on SRH and mortality outcomes. Because of the positive association of exercise and memory in the literature (16), we tested DWR at time 2 as a temporal mediator of the relationship between mild activity at time 1 and health at time 3. We tested DWR at time 2 as a mediator of the relationship between mild activity at time 1 and mortality. We also tested mild activity at time 2 as a temporal mediator of the relationship between depression at time 1 and mortality that has been proposed to explain the association of depression and mortality (9). To estimate bootstrap standard errors and confidence intervals (CIs) for total and mediating effects, we used an SPSS macro (17) that was based on *κ* being equal to 1,000 bootstrap samples and using 95% CIs for the mediated effect.

## Results

Sample characteristics are presented in Table 1. At baseline, a higher percentage of obese respondents reported chronic pain, high blood pressure, a heart condition, diabetes, and arthritis, stroke, cancer, and lung disease than nonobese participants (*P* < .05). At times 2 and 3 these differences remained, except that the prevalence of stroke and cancer did not differ between obese and nonobese respondents. Preliminary analyses tested the effects of sex and age on SRH and mortality. Only age was correlated with SRH and mortality. Correlations between the study’s risk and protective factors and SRH and mortality are presented in Table 2.

**Table 1:** Sample Characteristics of 17,930 Adults, Health and Retirement Study (HRS)

Characteristic^a^	Range	Mean (Standard Deviation)
Age, y	50–104	68.65 (10.53)
Years of education	0–17	12.37 (3.29)
Depression^b^	0–8	1.52 (2.00)
Sleep^c^	1–3	2.45 (0.75)
Mild activities^d^	1–5	3.37 (1.18)
Body mass index^e^	10.98–85.85	28.89 (6.05)
Delayed word recall^f^	0–10	4.35 (2.06)
Self-rated health^g^	1–5	3.11 (1.13)
Age at death, y	50–110	80.44 (10.20)

a Data for all characteristics except age at death are from 2006 HRS data.

b Measured with a sum of 8 items from the 8-item version of the Centers for Epidemiological Study, Depression Scale (CES-D). Higher numbers indicated more depressive symptoms.

c Measured using the question, “How often do you feel really rested when you wake up in the morning? Would you say most of the time, sometimes, or rarely or never?” Responses were 1 = rarely or never, 2 = sometimes, and 3 = most of the time.

d Assessed with 1 item that asked, “How often do you take part in sports or activities that are mildly energetic, such as vacuuming, laundry, or home repairs?” Responses were 1 = hardly ever or never, 2 = 1 to 3 times a month, 3 = once a week, 4 = more than once a week, and 5 = every day.

e Body mass index (kg/m^2^) was calculated from weight and height at time 1.

f The number of words recalled in any order from a list of 10 nouns.

g Self-rated health was measured with 1 item that asked, “Would you say your health is excellent, very good, good, fair, or poor?” Responses were 1 = poor, 2 = fair, 3 = good, 4 = very good, and 5 = excellent.

**Table 2:** Bivariate Association of Self-Rated Health and Mortality of 17,930 Adults, Health and Retirement Study (HRS), 2006, 2008, and 2010

Characteristic^a^	Self-Rated Health^b^						Mortality	
	Time 1		Time 2		Time 3			
	*R*	*P* Value^c^	*R*	*P* Value^c^	*R*	*P* Value^c^	*R*	*P* Value^c^
Age	−.165	<.001	−.126	<.001	−.123	<.001	NA	NA
Education	.317	<.001	.297	<.001	.283	<.001	−.076	.006
DWR	.253	<.001	.227	<.001	.231	<.001	−.345	<.001
Depression	−.425	<.001	−.367	<.001	−.342	<.001	−.029	.33
Sleep	.288	<.001	.242	<.001	.228	<.001	.044	.11
Mild activities	.297	<.001	.232	<.001	.205	<.001	−.180	<.001
Smoking	−.082	<.001	−.089	<.001	−.104	<.001	−.378	<.001
BMI	−.129	<.001	−.149	<.001	−.155	<.001	−.242	<.001

Abbreviations: NA, not applicable; DWR, delayed word recall; BMI, body mass index.

a Data for age, years of education, DWR, depression, sleep, mild activities, smoking, and BMI are from 2006 HRS data.

b Time 1, 2006 data; Time 2, 2008 data; Time 3, 2010 data.

c *P* values calculated using bivariate correlations; significance set at *P* < .05, 2-tailed.

The total variation in outcomes of SRH at times 1, 2, and 3, with all of the predictors, was calculated with 3 hierarchical multiple regressions. Age and years of education in model 1 accounted for significant variance in SRH at time 1 (9%), time 2 (8%), and time 3 (7%). DWR, depression, sleep, mild activities, smoking, and BMI were entered as a set and accounted for significant incremental variance in SRH at time 1 (20%), time 2 (16%), and time 3 (14%), independent of the effects of age and education. All variables explained unique variance when predicting SRH (Table 3). In the Cox regression analysis, SRH, DWR, depression, sleep, mild activities, smoking, and BMI were entered as a set, controlling for age and education (Table 4). Significant predictors of mortality included age, DWR, depression, smoking, and BMI.

**Table 3:** Multiple Linear Regression Analyses Predicting Self-Rated Health of 17,930 Adults, Health and Retirement Study (HRS), 2006, 2008, and 2010^a^

Characteristic	Self-Rated Health^b^					
	Time 1		Time 2		Time 3	
	β	*P* Value	β	*P* Value	β	*P* Value
**Model 1^c^**						
Age	−.063	<.001	−.049	<.001	−.049	<.001
Education	.286	<.001	.286	<.001	.249	<.001
**Model 2^c^**						
DWR	.090	<.001	.090	<.001	.103	<.001
Depression	−.276	<.001	−.240	<.001	−.219	<.001
Sleep	.148	<.001	.120	<.001	.117	<.001
Mild activities	.140	<.001	.115	<.001	.085	<.001
Smoking	−.074	<.001	−.081	<.001	−.094	<.001
BMI	−.120	<.001	−.118	<.001	−.126	<.001
Model 1 *R^2^*	.090	<.001	.077	<.001	.067	<.001
Δ*R^2^*	.204	<.001	.156	<.001	.140	<.001
*R^2^*	.294	<.001	.233	<.001	.207	<.001
*F* Model 1^d^	440.223	<.001	385.342	<.001	370.426	<.001
*F* Model 2^d^	463.205	<.001	290.346	<.001	227.575	<.001

Abbreviations: DWR, delayed word recall; BMI, body mass index.

a *P* values calculated using multiple and simple regression; significance set at *P* < .05.

b Time 1, 2006 data; Time 2, 2008 data; Time 3, 2010 data.

c Data for age, years of education, DWR, depression, sleep, mild activities, smoking, and BMI are from 2006 HRS data.

d *F* test for model.

**Table 4:** Cox Regression Analysis Predicting Survival of 17,930 Adults, Health and Retirement Study, 2006, 2008, and 2010

Factor	Hazard Ratio^a^ (95% CI)	*P* Value
Age	1.08 (1.07–1.09)	<.001
Education	0.99 (0.97–1.01)	.22
Self-rated health	0.28 (0.07–1.14)	.08
Delayed word recall	0.87 (0.85–0.90)	<.001
Depression	1.10 (1.07–1.13)	<.001
Sleep	0.96 (0.89–1.04)	.35
Mild activities	0.70 (0.32–1.56)	.39
Smoking	1.63 (1.41–1.89)	<.001
Body mass index	0.98 (0.97–0.99)	.001

Abbreviation: CI, confidence interval.

a Estimated with Cox proportional hazards regression while adjusting for other factors in the model.

Tests of temporal mediation were conducted. DWR at time 2 mediated the relationship between mild activities at time 1 and SRH at time 3. DWR at time 2 also mediated the relationship between mild activities at time 1 and mortality. Effect sizes were small (*κ* ^2^ = .03 for SRH and *κ* ^2^ = .04 for mortality). Because including DWR at time 2 would likely eliminate the sickest participants (who had died by time 2) in the mortality mediation model, we also tested DWR at time 1 as a mediator of mild activities at time 1 and mortality, which was significant. The effect size was stronger than the effect of DWR at time 2, but still small (*κ* ^2^ = .07). Mild activities at time 2 mediated the relationship between depression at time 1 and time 3 SRH; the effect size was small (*K* ^2^ = .04). Mild activities at time 2 mediated the relationship between depression at time 1 and mortality; the effect size was small (*K* ^2^ = .04). Depression at time 2 also mediated the relationship between mild activity at time 1 and SRH at time 3; the effect size was small (*K* ^2^ = .05) (Table 5).

**Table 5:** Indirect Effects of Self-Rated Health and Mortality of 17,930 Adults, Health and Retirement Study, 2006, 2008, and 2010

Indirect Effect With T1, T2, and T3 Characteristics	Point Estimate (SE)	Bootstrap 95% CI	Effect Size,^a^ κ^2^
T1 activity/T2 DWR/T3 SRH	0.0352 (0.0025)	0.0306 to 0.0402	.0342
T1 activity/T2 DWR/mortality	−0.3059 (0.0854)	−0.4753 to −0.1515	.0402
T1 activity/T1 DWR/mortality	−0.4967 (0.0820)	−0.6668 to −0.3549	.0649
T1 depression/T2 activity/T3 SRH	−0.1867 (0.0116)	−0.2086 to −0.1643	.0447
T1 depression/T2 activity/mortality	1.518 (0.3407)	0.9640 to 2.3708	.0401
T1 activity/T2 depression/T3 SRH	0.0470 (0.0036)	0.0407 to 0.0543	.0467

Abbreviations: SE, standard error; CI, confidence interval; DWR, delayed word recall; SRH, self-rated health; T1, time 1 (2006); T2, time 2 (2008); T3, time 3 (2010).

a We used an SPSS (SPSS Institute, Inc, Cary, North Carolina) macro to estimate bootstrap standard errors and CIs for total and mediating effects, based on *K* = 1,000 bootstrap samples and using 95% CIs for the mediated effect.

## Discussion

The purpose of this study was to examine predictors of perceived health and mortality in a nationally representative sample of older adults. This study measured self-rated health with a single item that is correlated with multiple physical health indicators (15) and is a quick and economical way to assess physical health. One-item, global self-ratings of health that ask people to rate their health as poor, fair, good, very good, or excellent are frequently used in medical and research settings and by the Centers for Disease Control and Prevention as a global self-rating of health-related quality of life in the annual Behavioral Risk Factor Surveillance System (18). A global self-rating of health may predict mortality independent of other medical, behavioral, or psychosocial factors (19). The rating is effective for assessing physical health and is highly predictive of mortality, perhaps partly because people evaluate their health using information about bodily sensations that are related to important physiological functions, such as inflammation (20).

Depression was the strongest predictor of SRH at time 1 and remained strong at all points. Older adults may not recognize symptoms of depression or may assume that there are physical causes for them, which may lead them to seek treatment from a primary health provider rather than a mental health professional (21,22). In addition to encouraging regular physical activity as part of prevention efforts in older adults, health providers should screen older adults for depression and refer them for further evaluation or treatment to prevent declining health.

BMI at time 1 was strongly correlated with SRH at each point. In regression analyses, BMI was also a significant predictor of mortality. The prevalence of chronic health conditions (pain, high blood pressure, heart condition, diabetes, and arthritis) differed between obese and nonobese participants at all points.

In mortality analyses, smokers were more likely to die than nonsmokers, which is consistent with previous research (3). DWR was another important predictor, which highlights the importance of assessing memory difficulties and creating effective interventions. Recent evidence indicates that cognitive training improved memory for 5 years or more in healthy individuals (23). More research is needed to test the effectiveness for people with memory impairments. We found that education, BMI, smoking, and mild activities helped explain variance in DWR, which is consistent with research indicating the importance of education and lifestyle factors for cognitive health. A review of predictors of cognitive change identified education, hypertension, health, cardiovascular disease, and the APOE e4 gene as significant predictors; however, effects of physical activity on cognitive change were inconclusive (24). Recent evidence suggests that memory may be malleable in late life. Higher levels of cognitive activity over the lifetime are associated with less cognitive decline, independent of neuropathologic burden (25), and a growing body of evidence indicates that aerobic exercise may increase hippocampal volume, lead to better memory function (26), slow cognitive decline, and reduce the risk of dementia (16). Cognitive training and cognitive stimulation have demonstrated improved attention, learning, and memory functioning in healthy older adults and those with dementia (27,28), and preliminary findings suggest that transcranial direct current stimulation (29) and deep brain stimulation may sustain or even improve memory functioning in early AD (30). Whether these efforts will translate to reduced mortality is unknown. These results suggest that health providers should screen for cognitive impairment, refer patients to mental health professionals for further evaluation, and recommend behaviors that promote cognitive health.

Tests of temporal mediation indicated that DWR at time 2 mediated the relationship between time 1 mild activities and time 3 SRH and between time 1 mild activities and mortality, suggesting that exercise positively influences memory, which then influences health and longevity. The mediating effect of DWR at time 1 was stronger, suggesting that the effects of exercise on memory may lessen with time. Mild activities mediated the relationship between depression and SRH, and between depression and mortality. Depression also mediated the relationship between mild activity and SRH. Together these results suggest a bidirectional causal relationship between exercise, depression, and health, such that activity reduces depression, which positively influences health. Similarly, depression may inhibit physical activity, which may then diminish health. Thus, it is important to assess and encourage frequent physical activity and to assess and treat depression (22), as these efforts may offer benefits on health through separate causal pathways.

Limitations of this study include the lack of genetic information and lack of corroboration with objective behavioral indicators. Future studies should consider experimental tests, daily experience sampling, and longer longitudinal follow-ups of risk and protective factors. Researchers should better understand the processes by which these risk and protective factors affect health. Additional longitudinal tests of mediation and moderation of risk and protective factors may help explain the specific causes of physical health and mortality. Overall, researchers should integrate demographic, biological, cognitive, and behavioral findings from multiple comprehensive longitudinal studies of aging to better understand the risk and protective factors for health and mortality. This integration in turn could lead to the development of more effective screening and interventions.
